# High-dose L-theanine–caffeine combination improves neurobehavioural and neurophysiological measures of selective attention in acutely sleep-deprived young adults: a double-blind, placebo-controlled crossover study

**DOI:** 10.1017/S0007114525104169

**Published:** 2025-08-14

**Authors:** Gayani S. Nawarathna, Dewasmika I. Ariyasinghe, Tharaka L. Dassanayake

**Affiliations:** 1 Department of Physiology, Faculty of Medicine, University of Peradeniya, Peradeniya, Sri Lanka; 2 Department of Basic Sciences, Faculty of Dental Sciences, University of Peradeniya, Peradeniya, Sri Lanka; 3 Department of Psychiatry, Faculty of Medicine, University of Peradeniya, Peradeniya, Sri Lanka; 4 Teaching Hospital Peradeniya, Peradeniya, Sri Lanka; 5 School of Psychological Sciences, The University of Newcastle, Newcastle, Australia

**Keywords:** Caffeine, L-Theanine, Driving, Selective attention, Electroencephalogram, Sleep deprivation, Reaction time, Event-related potentials

## Abstract

L-theanine, an amino acid found in tea, and caffeine, found in tea and coffee, are claimed to enhance attention. We conducted a double-blind, placebo-controlled, counterbalanced, two-way crossover trial to determine the acute effects of a high-dose L-theanine–caffeine combination on neurobehavioural (reaction time) and neurophysiological (P3b cognitive event-related potential (ERP)) measures of selective attention in acutely sleep-deprived healthy adults. Thirty-seven overnight sleep-deprived healthy adults (aged 22–30 years, twenty-one men) completed a computerised traffic-scene-related visual stimulus discrimination task before and 50 min after ingesting 200 mg L-theanine–160 mg caffeine combination or a placebo. The task involved selectively responding to imminent accident scenes (20 % probability) while ignoring randomly intermixed, more frequent safe scenes (80 % probability). A 32-channel electroencephalogram was recorded concurrently to derive ERP. The L-theanine–caffeine combination significantly improved the hit rate (*P* = 0·02) and target-distractor discriminability *(P* = 0·047), compared with the placebo. Although both L-theanine–caffeine combination (△ = 52·08 ms, *P* < 0·0001) and placebo (△ = 13·97 ms, *P* = 0·024) improved reaction time to accident scenes, the pre-post-dose reaction time improvement of the L-theanine–caffeine combination was significantly greater than that of placebo (△ = 38·1 ms, *P* = 0·003). Compared with the placebo, the L-theanine–caffeine combination significantly increased the amplitudes and reduced the latencies of P3b ERP component. Our findings suggest that L-theanine–caffeine combination improves the accuracy and speed of deploying selective attention to traffic scenarios in sleep-deprived individuals. This improvement is brought about by greater and faster neural resource allocation in the attentional networks of the brain.

In recent years, there has been growing interest in the use of food supplements to support cognitive performance. L-Theanine, also called γ-glutamylethylamide or γ-ethylamino-L-glutamic acid, is a non-protein-forming amino acid found specifically in the tea leaves (*Camellia sinensis*). Caffeine (1,3,7-trimethylxanthine) is present in coffee, tea and cocoa. A cup of tea contains 4–22 mg of L-theanine and 25–50 mg of caffeine, depending on the variety and brewing time^(1,2)^. Purified forms of L-theanine and caffeine are now available as nootropic supplements in the market.

The pharmacology of L-theanine and caffeine has been extensively studied. Ingested L-theanine is absorbed into the bloodstream through the small intestine via a sodium-coupled active transporter^(3,4)^. The peak plasma concentration of L-theanine occurs around 45–50 min after ingestion. It crosses the blood–brain barrier using a leucine-preferring transport system^(5)^.

L-Theanine is structurally similar to glutamic acid, the precursor of the neurotransmitter glutamate. Animal studies indicate that L-theanine is a low-affinity glutamate receptor partial agonist^(6)^. It also enhances the release and concentration of dopamine^(7,8)^ and serotonin^(9)^, which play crucial roles in various cognitive processes. High doses of L-theanine have been demonstrated to be safe in humans. No significant adverse effects were reported with doses of up to 450–900 mg in patients with generalised anxiety disorder^(10)^. Similarly, children with attention-deficit hyperactivity disorder who were administered 400 mg of L-theanine daily for 6 weeks reported no significant adverse effects^(11)^.

Caffeine is rapidly absorbed into the bloodstream through the intestine, with complete absorption within 45 min after ingestion^(12)^. Peak plasma caffeine concentration is observed in 1-hour post-ingestion^(13)^. Caffeine readily crosses the blood–brain barrier^(14)^. Caffeine is an adenosine receptor antagonist. It increases the dopaminergic and cholinergic transmission in the brain. It is recognised as a central nervous system stimulant that enhances alertness, vigilance and attention^(13,15)^. Caffeine is considered safe in moderate amounts in healthy adults, with no significant adverse effects. However, high doses (400 mg per day equivalent to about 5·7 mg/kg body weight for a 70 kg adult) can lead to adverse effects^(16)^, characterised by nervousness, irritability, insomnia, sensory disturbances, diuresis, arrhythmia, tachycardia, hyperventilation and gastrointestinal disturbances^(17)^. Both L-theanine and caffeine are completely cleared from the plasma within 24 h^(17,18)^.

The present study focuses on the effects of L-theanine–caffeine combination on selective attention. Selective attention is the process of allocating attention among relevant inputs, thoughts and actions while simultaneously ignoring irrelevant or distracting stimuli^(19,20)^. Selective attention is applied in daily activities and plays a critical role in time-constrained activities like driving: For instance, a driver who approaches a traffic intersection must focus on traffic lights while ignoring distractions like pedestrians standing on pavements and roadside billboards.

Purified L-theanine and caffeine have been administered in various doses to evaluate their effects on cognitive functions. Caffeine is widely recognised for its ability to enhance selective attention in a dose-dependent manner^(13,21)^. High-dose L-theanine improves neurobehavioural measures (reaction times and task accuracy) of selective attention^(22–26)^. For instance, Higashyama *et al.* ^(23)^ reported that 200 mg of L-theanine enhanced auditory reaction time in participants with higher anxiety propensity. Similarly, Kahathuduwa *et al.* ^(24,27)^ showed that the same dose of L-theanine improves recognition visual reaction time in healthy volunteers. High-dose L-theanine seems to enhance attentional processing in a dose-dependent manner^(25,26)^, yet the amount of L-theanine in a single cup of tea seems to be insufficient to significantly enhance attention^(24)^. Previous research also shows that the degree of attentional improvement caused by the L-theanine–caffeine combination is greater than the degree of improvement caused by either compound alone, suggesting a synergistic effect^(24,27–30)^.

Neural mechanisms underlying the attentional effects of L-theanine and caffeine have been investigated with electroencephalography (EEG), cognitive event-related potentials (ERP) and functional MRI. In a high-density EEG-mapping study, Gomez-Ramirez *et al.* ^(31)^ observed that a 250 mg dose of L-theanine induced a phasic increase in alpha power in brain regions associated with visual processing during task anticipation while suppressing background *α* activity. This was indicative of enhanced target–distractor discrimination. ERP are EEG signals evoked by a stimulus or neural process and can be extracted through filtering out background EEG activity and averaging the number of EEG epochs time locked to the event of interest. The P3b component, a late ERP marker of attentive processing, is widely used in cognitive psychophysiology. P3b is a positive deflection that peaks around 300 ms post-stimulus, with a prominent centro-parietal scalp distribution^(32–34)^. In a classic oddball paradigm, P3b appears in response to infrequent targets (i.e. oddball), but not for frequent standard stimuli. The key indices measured in P3b are its amplitude and latency: Amplitude refers to the difference between the mean pre-stimulus baseline voltage and the highest positive peak of the P3b ERP waveform. It is an index of the extent of neural resources allocated for attentional processing. P3b latency is the time elapsed from the onset of the stimulus to the point of the P3b peak. It is an index of the speed of attentional deployment and context updating. L-Theanine, caffeine and L-theanine–caffeine combination are known to elicit a larger P3b amplitude than placebo^(24)^. Additionally, high-dose L-theanine (400 mg) has been reported to significantly reduce P3b latency^(26)^. Functional MRI studies further elucidate the role of L-theanine and caffeine in enhancing selective attention. Kahathuduwa *et al.* ^(27)^ observed that L-theanine reduces blood oxygenated Hb level-dependent (BOLD) responses to distractor stimuli in brain regions governing visual attention, and both L-theanine and caffeine significantly decrease functional MRI responses in the right precuneus, a key region within the default mode network associated with mind wandering. This evidence suggests that the L-theanine–caffeine combination enhances attentional focus by suppressing mind wandering and deviation of attention to distracters^(27)^.

The research studies discussed above on the acute attentional effects of L-theanine–caffeine combination have primarily concentrated on healthy volunteers tested under optimal conditions, using abstract task paradigms. In everyday life, however, people consume L-theanine and caffeine often when they feel tired or sleep deprived and when they have to attend to demanding everyday activities like driving. Our recent work shows that high-dose L-theanine improves accuracy and reaction time in a traffic-related attention task in sleep-deprived healthy adults^(35)^. Given that the L-theanine–caffeine combination has greater attentional effects than L-theanine alone, we hypothesised that the L-theanine–caffeine combination may enhance attention more effectively in individuals experiencing sleep deprivation. To test this hypothesis, we investigated whether an orally administered combination of 200 mg of L-theanine and 160 mg of caffeine could enhance neurobehavioural measures (reaction time and response accuracy) and neurophysiological measures of selective attention in a traffic-scene-related visual recognition reaction task in sleep-deprived healthy young adults. To this end, we conducted a double-blind, placebo-controlled, counterbalanced, crossover trial, comparing the effect of the L-theanine–caffeine combination with that of a placebo. The primary neurophysiological outcome measure of interest in this study was the P3b ERP component, which is found to be sensitive to attentional deficits of sleep deprivation^(36,37)^ and attentional enhancement by L-theanine and caffeine^(24,26)^.

## Materials and methods

### Participants

A sample of thirty-six participants was required to observe an effect size (Cohen’s d) of 0·5^(38)^, with an *α* error of 0·05 and a power of 80 %. A sample size of 40 was decided upon, assuming a 10 % attrition rate. We screened a total of forty-one healthy young adults to assess their eligibility for the study, excluded one respondent who was colour blind and recruited forty young adults (age: 22–30, twenty-one males). All had normal or corrected-to-normal vision and normal hearing. None of the participants had significant neurological/psychiatric illnesses. None were on medications that have neurological effects, used alcohol regularly or used tobacco products or recreational psychoactive drugs. All participants consumed less than four cups of tea or coffee per day. The study received approval from the Research Ethics Committee of the Faculty of Medicine, University of Peradeniya and was conducted in accordance with the World Medical Association Declaration of Helsinki^(39)^. Informed written consent was obtained from all participants who met the eligibility criteria. Each participant was compensated with 3000 Sri Lankan rupees (10 USD) for their participation. The trial protocol has been registered in the Sri Lanka Clinical Trial Registry (registration number: SLCTR/2023/006, https://slctr.lk/trials/slctr-2023–006).

### Study design and treatment

This study was a double-blind, placebo-controlled, counterbalanced, two-way crossover trial. The study was conducted in the Clinical Neurophysiology and Cognitive Neuroscience (CLINCON) laboratory at the Department of Physiology, Faculty of Medicine, University of Peradeniya. We compared a high-dose L-theanine–caffeine combination (200 mg L-theanine + 160 mg caffeine) with a placebo (360 mg of starch) on their effects on reaction time measures and concurrently recorded ERP in a traffic-scene-based recognition visual reaction task. The rationale for selecting 200 mg of L-theanine and 160 mg of caffeine was based on previous experimental evidence and safety guidelines^(10,16,24,35,40)^. These selected doses are also consistent with the amounts found in many commercial nootropic supplements and functional beverages, thus increasing the ecological validity of the study. To minimise order effects, we counterbalanced the treatment sequence by assigning half of the participants to receive the active treatment first, followed by the placebo, while the other half received the treatments in the reverse order. L-Theanine and caffeine were obtained as a commercial preparation in 100 % purified powdered form (Green Labs Nutrition) and stored in airtight packaging prior to preparation. The specific doses of L-theanine (200 mg), caffeine (160 mg) and starch (360 mg) were measured with a digital microscale having a sensitivity of 1 mg. Each measured L-theanine–caffeine combination and placebo was incorporated into transparent gelatine capsules. The participants were asked to swallow the capsule with 100 ml of water. The two treatments were indistinguishable from one another, and we did not reveal the administered dose to the participants. One investigator (GN) conducted all the tests and processed EEG/ERP data. The capsules were dispensed by a third person, so the investigator was also blind to the treatment conditions.

### Tests and measurements

**Figure 1.**
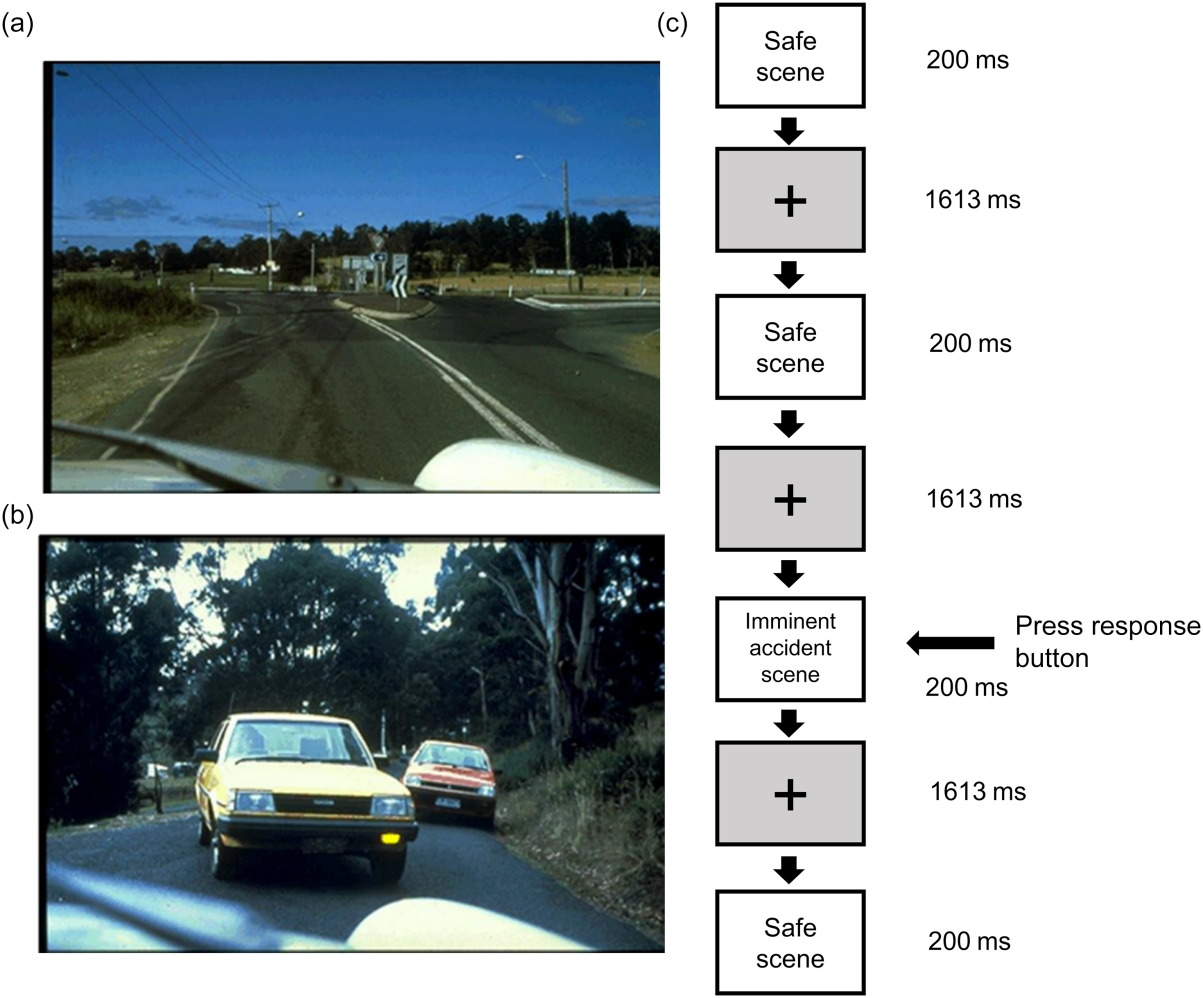
Traffic task stimuli and paradigm: (a). A sample of a safe traffic scene; (b). a sample of an imminent accident scene; (c). a sample sequence of the task paradigm.

We administered a traffic-scene-based visual oddball task originally developed by Martin *et al.* ^(41)^ in which the participant had to respond to imminent accident scenes while ignoring safe scenes (Figure 1). The task and its validity in psychopharmacology have been described in detail by the original authors^(41,42)^ and, more recently, by Karunaratne and Dassanayake^(35)^. Visual stimuli were presented on a standard 14·5” PC screen with a resolution of 1024 by 768 pixels, and the responses were recorded using the Presentation® (Neurobehavioural Systems, Inc.) software on a Windows^TM^-based personal computer. The participants performed the task in a seated position, fixating their eyes at the centre of the screen placed at a distance of 60 cm. Each visual stimulus, a 384-by-256-pixel traffic scene as a car driver would see through the windscreen, was presented in the centre of the screen for 200 ms. The stimuli were presented with an inter-stimulus interval of 1613 ms. There was a 20 % probability for a given stimulus to be an imminent accident scene (i.e. target stimulus) and an 80 % probability for it to be a safe (non-accident) traffic scene (i.e. standard stimulus). The participants were holding the press pad and pressed the response button as fast as they could with the thumb of their dominant hand for the targets while ignoring the standard stimuli. A total of 450 stimuli (90 targets and 360 standards) were presented throughout the task in two blocks, each block consisting of forty-five targets and 180 standards randomly intermixed within the task block to prevent participants from predicting their sequence. Each task block was run for about 7 min, and the whole task took about 15 min.

Concurrent EEG was recorded during the task to derive ERP and study the underlying neurophysiological processes. A BioSemi Active Two EEG/ERP acquisition system with ActiView software (BioSemi) was used to acquire EEG data. Continuous EEG data were acquired from a thirty-two-channel electrode cap configured to international 10–20 scalp electrode placement standards^(43)^, with the placement of two pairs of additional horizontal and vertical electrodes for electro-oculography, and two electrodes over left and right mastoids (M1 and M2) for re-referencing. During the acquisition of data, the EEG signals were digitised at a rate of 1024 Hz. Electrode offset at each site was maintained within ±40 mV. The high-pass filter was set at 0·1 Hz and the low-pass filter at 30 Hz. EEG data were recorded using common average referencing in the BioSemi Active Two system. A detailed description of this electrode referencing system can be found on the product website (https://www.biosemi.com/faq/cms&drl.htm).

After acquiring the continuous EEG data, subsequent stages of EEG data processing were done offline using an automated script in EEG Display version 6.4.11 (Functional Neuroimaging Laboratory, University of Newcastle): Bipolar horizontal (HEOG) and vertical EOG (VEOG) channels were generated using the eye electrode recordings. Active EEG site recordings were re-referenced to the average of the left and right mastoids. Continuous EEG recordings were subjected to blink artifact reduction, creating a blink model using the VEOG recordings and epoched (–100 to 1000 ms) time-locked to stimulus (i.e. target or standard) onset. All epochs with voltage deviations beyond ± 40 μV were rejected, and the rest were baseline-corrected and averaged separately for standard and target trials. Automatic peak detection was performed on averaged ERP waveforms to measure amplitudes and latencies. In each averaged ERP recording, we concentrated on the attentive component (P3b) of ERP. The P3b component was extracted by isolating the maximum positive peak in a latency window of 280–600 ms.

### Testing protocol

**Figure 2.**
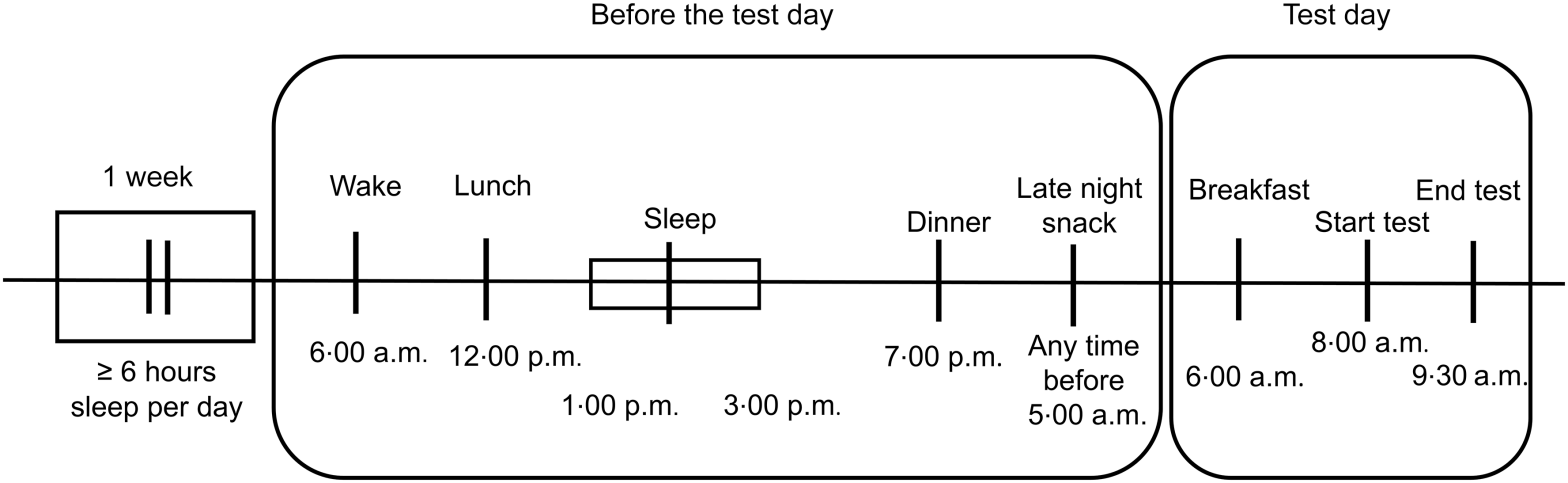
Test preparation timeline.

The testing protocol has been adapted from one that we used recently to study the attentional effects of L-theanine on sleep-deprived individuals^(35)^. The test preparation timeline commenced 8 d before testing (Figure 2). Participants were instructed to get at least 6 h of sleep per night for the first 7 d before the experiment. Participants abstained from alcohol for at least 48 h and tea, coffee or other caffeinated beverages for 24 h before testing. On the day before testing, the participants continued their daily activities as usual and had a 2-hour siesta after lunch. After this, the participants stayed awake for 17 h until the testing session on the following day. The research team maintained frequent contact with the participants to ensure that they were strictly following the above instructions. Testing was performed in the mornings, starting at 08.00.

**Figure 3.**
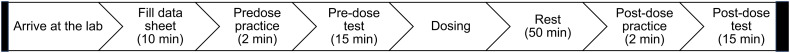
Testing and treatment protocol for a given day of testing.

The daily testing protocol is outlined in Figure 3. Upon arrival at the laboratory on each testing day, participants were assessed for compliance with the preparation guidelines, and they were queried about their subjective feeling of sleepiness. Then the participants were allowed to relax for 10 min. Participants were then introduced to the traffic task, in which they were instructed on the importance of both speed and accuracy of responses. Initially, participants underwent pre-dose testing, which involved performing the traffic task while EEG recordings were taken. This consisted of a 2-minute practice session followed by the actual task, which lasted approximately 15 min. Subsequently, they consumed the treatment prepared for the session and remained seated for 50 min, watching a non-stimulating wildlife documentary. Following this, participants did the post-dose testing, which was the same as the pre-dose test. The interval between two testing days ranged from 3 to 7 days. On the second testing day, participants followed the same protocol, but with alternative treatment.

### Data analysis

The data of all participants (across two counterbalanced arms) were pooled together for data analysis. The neurobehavioural outcome measures were hits (correct responses to targets, i.e. imminent accident scenes), misses (missed responses to targets), false alarms (active responses made for safe scenes) and mean hit reaction time (time from onset of the target stimulus to the response). Additionally, A′ (A prime) – a non-parametric measure of target-distractor discriminative sensitivity – was calculated based on the probability of hits and false alarms^(44)^. In this paradigm, A’ reflects an individual’s ability to accurately distinguish between imminent accident scenes from safe scenes. Higher A’ values indicate better discrimination performance. The neurophysiological outcome measures were the peak amplitudes and latencies of the P3b ERP component in predetermined centro-parietal CZ, CP1, CP2, PZ, P3 and P4 scalp sites, where the P3b ERP is expected to be most prominent^(34)^.

The accuracy measures (viz. probability of hits, probability of false alarms and A′) had highly skewed distributions and were analysed using the Wilcoxon signed rank test. The hit reaction time and P3b EPR latencies and amplitudes were normally distributed, and their variances across treatment and time conditions were similar, conforming to the normality and homoscedasticity assumptions. Consequently, treatment (L-theanine and placebo) × time (pre-dose and post-dose) two-way, within-subject ANOVA tests were conducted to analyse the treatment and time effects on hit reaction time, P3b ERP amplitude and P3b latency. Subsequent pairwise pre- *v*. post-dose comparisons of group means (i.e. change from baseline) of hit reaction times and latencies and amplitudes of P3b ERP for each treatment were performed, using paired *t* tests. The level of significance was set at a *P* value of 0·05. Given the active treatment *v*. placebo comparison and the P3b scalp sites were determined *a priori* based on the study hypothesis, we did not adjust the *P* values for multiple comparisons. All the statistical analyses were done using Statistical Package for the Social Sciences (SPSS™) version 21.0. (IBM Corp.).

## Results

### Participant characteristics

**Figure 4.**
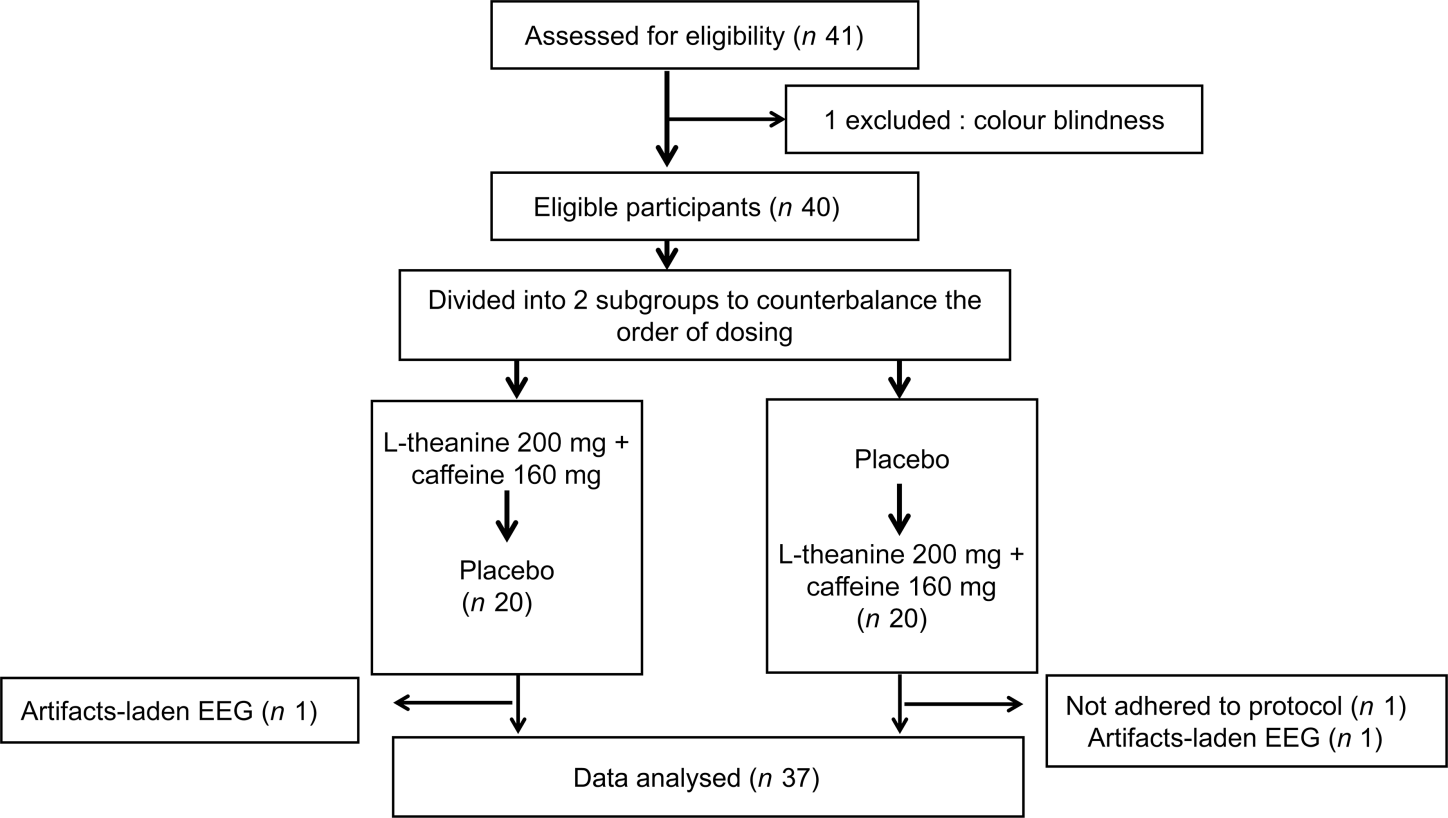
CONSORT diagram of participant recruitment and testing.

Out of the forty eligible participants, three were excluded after allocation (Figure 4): One was excluded since the participant did not adhere to the test preparation protocol. The second participant blinked too often during the test sessions, and the EEG data were contaminated with too many eye-blink artefacts. The third participant’s data were excluded from the analysis due to contamination of the EEG recording with excessive drift artefacts. The data of the remaining thirty-seven overnight-sleep-deprived healthy young adults (mean age = 25·86, sd = 2·21, 21 males) were analysed. Thirty-four participants were right handed and three were left handed on self-report.

### Neurobehavioural data

The main neurobehavioural outcome measures were pre- and post-dose hits, false alarms, target-distractor discrimination sensitivity (A’) and hit reaction time. Table 1 summarises the results of the accuracy data. A significant improvement in the probability of hits (*P* = 0·005) and A′ (*P* = 0·008) was observed with the L-theanine–caffeine combination. No significant changes in either the probability of hits (*P* = 0·881) or A′ (*P* = 0·762) were observed with the placebo. There was a significant improvement in the probability of hits (*P =* 0·02*)* and A’ (*P* = 0·047) by the L-theanine–caffeine combination compared with the placebo. Neither the L-theanine–caffeine combination nor the placebo significantly changed the false alarm rate (*P* > 0·05).

**Table 1.** Pre- and post-dose comparison of hits and discrimination sensitivity (A′) between L-theanine–caffeine combination and placebo

Treatment	Pre-dose		Post-dose		Significance (*P)* ^*^
	Median	IQR	Median	IQR	
Hits					
T + C	88·0	84·0–90·0	90·0	89·0–90·0	0·005
Placebo	89·0	86·5–90·0	89·0	86·5–90·0	0·881
A′					
T + C	0·991	0·978–0·996	0·996	0·988–0·999	0·008
Placebo	0·992	0·984–0·997	0·994	0·982–0·997	0·762

IQR, interquartile range; T + C, L-theanine–caffeine combination.

* *P* for within-group comparison reported based on Wilcoxon Signed Rank test.

There was a significant treatment × time interaction of hit reaction time (*F*_(1,36)_ = 9·84, *P* = 0·003), indicating that the degree of improvement of reaction time was different between treatments. Pre-dose *v*. post-dose comparison of the individual treatments showed significant improvements in the hit reaction time by both L-theanine–caffeine combination (△ = 52·08 ms, *t* = 4·802, *P* < 0·0001) and placebo (△ = 13·97 ms, *t* = 2·365, *P* = 0·024) (Table 2). However, the mean hit reaction time improvement of the L-theanine–caffeine combination was significantly greater than that of placebo (△ = 38·1 ms, 95 % CI: 13·48, 62·73, *P* = 0·003).

**Table 2.** Comparison of the hit reaction time between the L-theanine–caffeine combination and the placebo

Treatment	Pre-dose		Post-dose		Mean RT improvement		
	Mean hit RT (ms)	sd	Mean hit RT (ms)	sd	Time (ms)	CI (95 %)	Significance (*P*)^*^
T + C	485·92	75·29	433·83	40·41	52·08	30·08, 74·08	< 0·001
Placebo	478·82	91·74	464·84	91·55	13·97	1·99, 25·96	0·024

RT, reaction time; ms, millisecond; CI, confidence interval; T + C, L-theanine–caffeine combination.

* *P* for within-group comparison reported based on a paired sample *t* test.

### Visual event-related potentials

**Figure 5.**
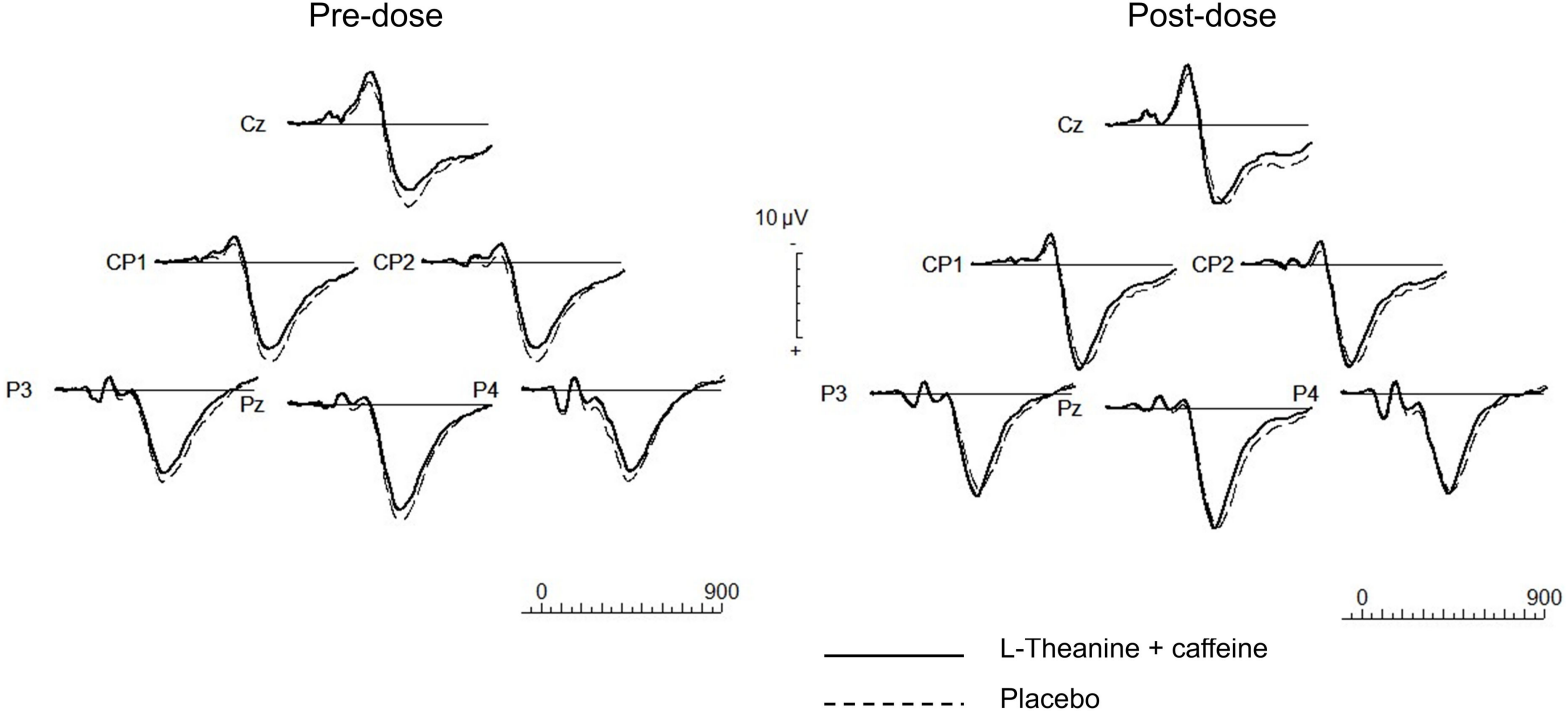
Grand average ERP waveforms.

Figure 5 shows the grand average visual ERP waveforms recorded at centro-parietal electrodes (CZ, CP1, CP2, PZ, P3 and P4 scalp sites) derived from target stimuli after L-theanine–caffeine combination and placebo treatments.

For the ERP data, the treatment × time ANOVA results are presented in Tables 3 and 4, and the pre- *v*. post-dose paired *t* test results are presented in Tables 5 and 6. There was a significant treatment × time interaction for the P3b ERP latency at all centro-parietal electrodes of interest (Table 3): L-theanine–caffeine combination significantly reduced P3b latencies in all scalp sites of interest, whereas the placebo did not change the P3b latencies in any site of interest (Table 5). The improvement of P3b latencies by L-theanine–caffeine combination was around 30 ms and was significantly greater than that of the placebo at each scalp site of interest (CZ: △ = −29·16, 95 % CI (–43·74, −14·58), *P* < 0·001; CP1: △ = −28·74, 95 % CI (–43·39, −14·08), *P* < 0·001; CP2: △ = −27·58, 95 % CI (–43·90, −11·25), *P* = 0·002; PZ: △ = −30·22, 95 % CI (–48·14, −12·29), *P* = 0·002; P3: △ = −28·29, 95 % CI (–46·03, −10·55), *P* = 0·003; P4: △ = −34·78, 95 % CI (–54·03, −15·53), *P* = 0·001).

**Table 3.** Summary results of two-way ANOVA on treatment (L-theanine–caffeine combination and placebo) and time (pre-dose and post-dose) effects on latency of visual P3b ERP in centro-parietal scalp positions

Scalp site	Time				Treatment × Time interaction			
	*F*	df	*P*	Effect size	*F*	df	*P*	Effect size
CZ	20·814	1,36	< 0·001	0·366	16·458	1,36	0·0002	0·314
CP1	18·272	1,36	< 0·001	0·337	15·826	1,36	0·0003	0·305
CP2	18·534	1,36	< 0·001	0·34	11·740	1,36	0·002	0·246
PZ	9·223	1,36	0·004	0·204	11·692	1,36	0·002	0·245
P3	16·749	1,36	< 0·001	0·318	10·466	1,36	0·003	0·225
P4	9·513	1,36	0·004	0·209	13·432	1,36	0·001	0·272

df, degree of freedom.

*P* for within-group comparison reported based on a paired sample *t* test.

**Table 4.** Summary results of two-way ANOVA on treatment (L-theanine–caffeine combination and placebo) and time (pre-dose and post-dose) effects on amplitudes of visual P3b ERP in centro-parietal scalp positions

Scalp site	Time				Treatment × Time interaction			
	*F*	df	*P*	Effect size	*F*	df	*P*	Effect size
CZ	1·690	1,36	0·202	0·045	8·402	1,36	0·006	0·189
CP1	5·358	1,36	0·026	0·13	7·298	1,36	0·01	0·169
CP2	4·060	1,36	0·051	0·101	4·937	1,36	0·033	0·121
PZ	2·808	1,36	0·102	0·072	1·671	1,36	0·204	0·044
P3	7·152	1,36	0·011	0·166	8·127	1,36	0·007	0·184
P4	9·990	1,36	0·003	0·217	2·562	1,36	0·118	0·066

df, degree of freedom.

*P* for within-group comparison was reported based on a paired sample *t* test.

**Table 5.** Summary of P3b latency paired *t* test data for L-theanine–caffeine and placebo

Scalp site	Treatment	Pre-dose		Post-dose		Mean latency difference		
		Mean latency (ms)	sd	Mean latency (ms)	sd	Latency (ms)	CI (95 %)	Significance (*P*)^*^
CZ	T + C	492·29	52·15	459·24	45·09	–33·04	–45·73, −20·35	< 0·001
	Placebo	486·9	52·82	483·02	48·99	–3·88	–12·82, 5·06	0·385
CP1	T + C	480·97	50·56	448·24	40·27	–32·72	–45·43, 20·02	< 0·001
	Placebo	476·37	48·86	472·39	50·27	–3·98	–13·86, 5·89	0·419
CP2	T + C	476·72	50·19	442·98	41·01	–33·73	–46·96, −20·49	< 0·001
	Placebo	476·16	48·64	470·01	47·62	–6·14	–17·75, 5·45	0·29
PZ	T + C	468·09	50·43	438·08	41·61	–30	–42·40, −17·61	< 0·001
	Placebo	459·19	44·57	459·4	43·6	0·21	–14·10, 14·52	0·976
P3	T + C	462·09	54·52	426·7	40·82	–35·39	–50·00, −20·78	< 0·001
	Placebo	455·47	53·09	448·37	47·7	–7·09	–19·97, 5·77	0·271
P4	T + C	461·99	46·44	431·27	43·28	–30·72	–18·25, −4·99	< 0·001
	Placebo	447·39	51·95	451·46	52·14	4·06	–9·48, 17·61	0·547

ms, milliseconds; SD, standard deviation; CI, confidence interval; T + C, L-theanine–caffeine combination.

* *P* for within-group comparison reported based on a paired sample *t* test.

**Table 6.** Summary of P3b amplitude paired *t* test data for L-theanine–caffeine and placebo

Scalp site	Treatment	Pre-dose		Post-dose		Mean amplitude improvement		
		Mean amplitude (µv)	sd	Mean amplitude (µv)	sd	Amplitude (µv)	CI (95 %)	Significance (*P*)^*^
CZ	T + C	9·88	5·62	11·56	5·81	1·67	0·35, 3·00	0·014
	Placebo	11·7	5·44	11·46	5·16	–0·24	–1·53, 1·05	0·707
CP1	T + C	12·25	5·65	14·04	5·97	1·78	0·69, 2·88	0·002
	Placebo	13·63	5·84	13·93	6·01	0·29	–0·75, 1·34	0·569
CP2	T + C	11·9	5·35	13·56	5·42	1·65	0·39, 2·91	0·011
	Placebo	13·36	5·28	13·53	5·39	0·16	–0·84, 1·18	0·741
PZ	T + C	14·25	6·32	15·47	6·18	1·21	0·04, 2·38	0·041
	Placebo	15·5	6·13	15·8	6·65	0·29	–0·86, 1·46	0·609
P3	T + C	11·74	5·1	13·56	5·64	1·81	0·76, 2·86	0·001
	Placebo	12·63	5·36	12·92	5·72	0·29	–0·58, 1·16	0·503
P4	T + C	11·13	5·01	13·07	5·46	1·93	0·63, 3·23	0·005
	Placebo	12·31	5·32	13·04	5·03	0·73	–0·24, 1·70	0·136

µv, microvolt; SD, standard deviation; CI, confidence interval; T + C, L-theanine–caffeine combination.

* *P* for within-group comparison reported based on a paired sample *t* test.

The treatment × time interactions were significant for the P3b amplitude in CZ, CP1, CP2 and P3 scalp sites (Table 4). Compared with the placebo, the L-theanine–caffeine combination significantly improved the P3b ERP amplitudes (Table 6) in those sites (CZ: △ = 1·92, 95 % CI (0·57, 3·26), *P* = 0·006; CP1: △ = 1·49, 95 % CI (0·37, 2·61), *P =* 0·01; CP2: △ = 1·49, 95 % CI (0·13, 2·85), *P* = 0·033; P3: △ = 1·52, 95 % CI (0·43, 2·60), *P* = 0·007).

It is noteworthy that, unlike classic waveforms generated by auditory oddball paradigms, we have observed in previous experiments^(26)^, early pre-attentive components (N1) were not consistently visible in these visual ERP recordings.

## Discussion

In this placebo-controlled crossover trial, we examined the acute effects of a high-dose L-theanine–caffeine combination on the accuracy and speed of selective attention deployment and the underlying neurophysiological processes. Previous studies have investigated the effects of the L-theanine–caffeine combination on attention in healthy adults tested under optimal conditions, yet there is limited evidence of how they affect sleep-deprived individuals – a condition in which beverages with L-theanine and caffeine are often consumed.

Previous studies show that L-theanine–caffeine combination can effectively enhance accuracy in attentional tasks^(22,28–30)^. In line with these findings, we observed that the combination of L-theanine and caffeine significantly enhanced the traffic task accuracy, improving the number of responses to imminent accident scenes and the ability to discriminate accident scenes from safe scenes.

Our recent work showed that L-theanine (200 mg) alone improves the discriminability of imminent accident scenes from safe scenes, along with a modest improvement in reaction time (∼20 ms) in acutely sleep-deprived young adults^(35)^. The improvements in reaction time are similar in healthy adults who underwent a visual colour discrimination task after the same dose of L-theanine (200 mg) and a comparable dose of caffeine (160 mg) alone. However, the current study demonstrates that L-theanine–caffeine combination shows a more prominent improvement in reaction time – around 40 ms. This is in the range of recognition visual reaction time improvement by L-theanine–caffeine combination we observed in a visual colour discrimination task in healthy volunteers previously^(24)^. These findings in combination suggest the presence of a synergistic effect in L-theanine–caffeine combination improving attention.

The functional neuroimaging and ERP findings suggest possible neural mechanisms underpinning the above attentional improvement. Kahathuduwa *et al.* ^(27)^ report that in healthy adults tested under optimal conditions in a visual selective attention task, L-theanine decreases functional MRI responses in visual information processing regions to distractor stimuli, whereas both L-theanine and caffeine decrease functional MRI responses to targets in brain areas that constitute default mode network that are involved in mind wandering^(27)^. Both of these are rather indirect mechanisms that enhance attention, without directly enhancing the target processing. In ERP, the P3b component is widely regarded as an index of attentional resource allocation elicited by task-relevant stimuli in oddball paradigms. Neurophysiological evidence suggests that P3b is generated when temporo-parietal networks, particularly the posterior parietal cortex, engage in evaluating the contextual significance of incoming stimuli for memory storage^(34,45)^. This process reflects top–down attentional control mechanisms that are thought to involve interactions between the parietal cortex and medial temporal lobe structures. The P3b is also sensitive to task demands and stimulus evaluation speed, and its latency is often used as a marker of stimulus classification efficiency^(34)^. In this context, our behavioural and concurrently recorded P3b ERP data show the improvement of selective attention in sleep-deprived conditions is brought about through more direct mechanisms: viz. through faster neural resource allocation (by about 30 ms, as indexed by improvement of P3b latency) and allocation of more neural resources (as indexed by larger P3b amplitude) for target processing. Further studies are necessary to elucidate how these direct and indirect attentional mechanisms are deployed depending on the task difficulty and the conditions under which the participants are tested.

Our findings have limitations in their generalisability. Though we used a traffic-related reaction task, the cognitive architecture of real–life driving is more complex and involves multiple operational-, tactical- and strategic-level processes^(46)^. To this end, our experimental design can be extended in the future to determine the effects of L-theanine–caffeine combination on standardised on-the-road driving tests^(47,48)^ and performance in driving simulators^(49)^, which previous researchers have used to determine the effects of different psychopharmacological agents. It should also be noted that chronic sleep deprivation may lead to more profound deficits in attention, and habitual use of L-theanine and caffeine may result in diminished attentional benefits over time due to the development of tolerance. Therefore, present findings cannot be directly extrapolated to those who have chronic sleep deprivation and/or habitually use high-dose L-theanine and caffeine.

In conclusion, our findings demonstrate that a high dose of the L-theanine–caffeine combination significantly enhances the accuracy and speed of selective visual attention in acutely sleep-deprived individuals performing traffic-related reaction tasks. This seems to occur through faster neural processing and increased neural resource allocation in the attentional circuits of the brain. Translating these findings to real-life driving scenarios, a high-dose L-theanine–caffeine combination may help acutely sleep-deprived drivers to execute quicker and more accurate responses to potential accident situations.
